# Characteristics and Screening Strategies of Hepatitis B in Guangdong Province, China

**DOI:** 10.3390/v18050486

**Published:** 2026-04-22

**Authors:** Weizhao Lin, Xiaoping Shao, Junjie Wang, Hongqing Wen, Jiahong Liu, Can Xiong, Zixia Qian, Wei Zhao, Jun Liu, Jiufeng Sun

**Affiliations:** 1 School of Public Health, Southern Medical University, Guangzhou 510515, China; 2 Guangdong Provincial Center for Disease Control and Prevention, Guangzhou 511430, China; 3 School of Public Health, Sun Yat-Sen University, Guangzhou 510080, China; 4 Department of Public Health and Preventive Medicine, School of Medicine, Jinan University, Guangzhou 510632, China; 5 School of Public Health, Guangdong Pharmaceutical University, Guangzhou 510310, China; 6 Guangdong Provincial Institute of Public Health, Guangzhou 511430, China

**Keywords:** HBsAg, prevalence, RDT, NAT, cost–benefit analysis

## Abstract

Determining the characteristics of hepatitis B virus (HBV) infection in the healthy population and evaluating the effectiveness of detection strategies will facilitate the optimization of hepatitis B screening strategies in the community and accelerate the elimination of HBV infection in China by the end of 2030. Hepatitis B surface antigen (HBsAg)-electrochemiluminescence immunoassays (ECLIAs), HBsAg-rapid diagnostic tests (RDTs), and HBV DNA-nucleic acid tests (NATs) were performed on serum samples from 2721 community-based healthy participants in Guangdong Province. The screening performance of the RDT and NAT and the distribution characteristics of HBsAg and HBV DNA were evaluated. The prevalence rates of HBsAg-ECLIA, HBsAg-RDT and HBV DNA-NAT in Guangdong Province were 6.10% (95% CI: 5.26~7.06), 4.96% (95% CI: 4.21~5.84) and 6.55% (95% CI: 5.64~7.49), respectively, and the prevalence rates for the three methods for individuals aged over 30 years were 11.18%, 10.92% and 12.57%, respectively. When the ECLIA was used as the gold standard, the sensitivities of the RDT, NAT and RDT and NAT in parallel were 80.7% (95% CI: 73.9~86.4), 86.7% (95% CI: 80.6~91.5) and 93.4% (95% CI: 88.5~96.6), respectively, and the sensitivity of the RDT and NAT in parallel was greater than that of the RDT alone (*p* < 0.001). The parallel RDT and NAT revealed an additional cost–benefit ratio (ACBR) < 1 for males and individuals aged over 30 years, which indicated that switching from the RDT screening strategy to the RDT and NAT in parallel is more cost effective. Adults aged over 30 years are the main population with hepatitis B infection in Guangdong Province, China, whose prevalence of HBsAg-ECLIA was 11.18%. Single RDT screening is prone to miss individuals with low levels of HBsAg. It is recommended to implement an RDT and NAT in parallel for individuals older than 30 years.

## 1. Introduction

Hepatitis B virus (HBV) infection is a major global public health challenge; in particular, in China, approximately 70 million HBV-infected individuals were diagnosed in 2017 [1,2,3,4]. As the province with the greatest HBV disease burden in China, Guangdong reported 2,213,954 hepatitis B cases from 2005 to 2022, with an HBsAg prevalence of 5.7% in the general population from 2006 to 2021 [5,6]. To permanently prevent hepatitis B threats, the World Health Organization (WHO) launched a global plan for hepatitis B elimination, in which the rates of hepatitis B incidence reduction, diagnosis and treatment and three-dose hepatitis B vaccine coverage among children under age 5 are expected to reach 90% by 2030 [7]. In 2020, three-dose vaccination coverage among children under age 5 in China reached 100%, which greatly exceeded the goal of the WHO. Unfortunately, the number of hepatitis B cases was 1.23 million, with a diagnostic rate of 58.78% in 2020, which is far from the goal of the WHO [8]. Therefore, first, it is urgent to implement screening in community-based healthy populations to fill this gap and achieve the 90% screening goal of the WHO by 2030.

To accelerate the elimination of hepatitis B, the Chinese government launched the Action Plan for the Prevention and Control of Viral Hepatitis during the period 2025~2030 in 2025. The plan aims to maintain a hepatitis B vaccine coverage rate of more than 95% for children, reduce the HBsAg prevalence rate to 0.2% for those under 5 years of age, and achieve a diagnosis rate of more than 80% for chronic hepatitis B cases among the general population [9]. Currently, HBsAg or HBV DNA positivity is used as a biological marker for HBV infection. In accordance with guidelines from the WHO and the U.S. Centers for Disease Control and Prevention (CDC), laboratory serological immunoassays are the preferred technique for detecting HBsAg. In resource-limited areas, rapid diagnostic tests (RDTs), such as the immune colloidal gold technique, are recommended as the primary screening approach for hepatitis B. For HBsAg seropositive individuals, nucleic acid tests (NATs), such as quantitative polymerase chain reaction (qPCR), should be used to measure the viral load [10,11,12].

Currently, there are two major challenges in both methodology and health economics when large-scale population screening is implemented. Laboratory serological immunoassays, such as electrochemiluminescence immunoassays (ECLIAs), are highly sensitive but costly and are difficult to implement in the general population. Rapid diagnostic tests (RDTs) are convenient and affordable, but their sensitivity is lower than that of laboratory serological immunoassays, which leads to missed detection of certain HBsAg-positive individuals [13]. In particular, laboratory serological immunoassays and RDTs are unable to detect HBsAg-negative HBV infection, also known as occult HBV infection (OBI), which has gradually become one of the critical challenges in hepatitis B screening [14]. NATs are highly sensitive for detecting HBV DNA, but they may miss HBV-infected individuals who are DNA negative and are relatively costly compared with RDTs [8,15,16]. Nevertheless, with numerous medical institutions possessing the ability to extract and detect nucleic acids and cost reductions driven by centralized procurement, NATs have the potential to become screening methods for hepatitis B [17,18]. Furthermore, the sensitivity of conducting RDTs and NATs in parallel is greater than that of conducting either an RDT or NAT alone [19,20]. Given that current guidelines do not recommend the NAT as a primary screening approach for the general population, studies on the performance and cost–benefits of NATs alone and RDTs and NATs in parallel in screening the general population are limited, and evidence is needed to evaluate the performance and cost benefits of RDTs, NATs, and RDTs and NATs in parallel in the general population [21].

In this study, the performance and cost–benefit of RDTs, NATs, and RDTs and NATs in parallel in the real world were evaluated, with laboratory serological immunoassays as the gold standard. Additionally, this study analyzed the characteristics of HBsAg and HBV DNA distributions, which is intended to provide evidence for formulating a graded, precision-based hepatitis B elimination screening strategy in Guangdong, China.

## 2. Method

### 2.1. Study Design

This was a cross-sectional study with multistage sampling and was conducted from May to December 2024. In accordance with the 2025 Guangdong Statistical Yearbook (https://stats.gd.gov.cn/gdtjnj/index.html, accessed on 13 January 2026), the gross domestic product (GDP) of 21 cities was ranked and categorized into three levels: high, medium, and low. High-GDP cities included Shenzhen, Guangzhou, Foshan, Dongguan, Huizhou, Zhuhai, and Jiangmen; medium-GDP cities included Maoming, Zhongshan, Zhanjiang, Shantou, Zhaoqing, Jieyang, and Qingyuan; and low-GDP cities included Shaoguan, Yangjiang, Shanwei, Meizhou, Chaozhou, Heyuan, and Yunfu. One representative city, including Guangzhou, Qingyuan, and Heyuan, was randomly selected from each GDP level. Within each selected city, one district or county was chosen randomly. After identifying the target districts and counties, we selected research participants through systematic sampling. First, we requested that the local district and county officials provide a list of permanent residents. Afterward, based on this list, we stratified the population into the following age groups: 0, 1~4, 5~14, 15~29, and 30 years and older. To ensure that the sample size for this study reached the minimum required level and remained balanced across age groups after stratification, we increased the sample size for individuals aged 15~29 and 30 years and above. We conducted systematic sampling for each age group on the list, selecting one participant for every 10 people. Finally, after finalizing the new list, we confirmed whether each subject was willing to participate in the survey via telephone or by visiting their residence. If they were willing, we asked them to visit the local Grade II Level A or higher hospital within three days for sample collection and to provide personal information. If sample collection and information gathering were not completed within three days, we excluded that subject to minimize time-related misclassification bias. China incorporated the hepatitis B vaccine into its National Immunization Program in 1992 and revised the program again in 2008. Consequently, individuals born before 1992 (aged 30 years and above) rarely received the hepatitis B vaccine, resulting in a very high prevalence of HBsAg. Among those born between 1992 and 2008 (aged 15–30), some received the hepatitis B vaccine, leading to a decrease in HBsAg prevalence. Among those born after 2008 (aged 0–14), nearly all received the hepatitis B vaccine, and the prevalence of HBsAg has remained low. To ensure an adequate sample size for the younger age groups (aged 0 and 1–4), we stratified our sample into the following age groups: 0, 1~4, 5~14, 15~29, and 30 years old and older [3]. The final sample sizes for the groups aged 0, 1~4, 5~14, 15~29, and 30 years old and above were 311, 381, 264, 675, and 1090, respectively. This study was reviewed and approved by the Ethics Committee of the Guangdong Provincial Center for Disease Control and Prevention. All the research subjects or their guardians signed informed consent forms. For the sample size of the study, we referred to Ahmed’s study to calculate the minimum sample size required to evaluate screening strategies using the following formula [22]:

$$n=\frac{Z_{1-\alpha }^{2}\times S_{N}\times (1-S_{N})}{\delta^{2}\times p}$$

where n represents the minimum sample size for the study, α represents the significance level and is set to 0.05, and $S_{N}$ represents the sensitivity of the screening method. The sensitivity of RDTs is typically lower than that of NATs and ECLIAs, which indicates that evaluating the screening performance of RDTs requires a large sample size; therefore, we used the sensitivity of the RDT to calculate the sample size. In accordance with Amie’s research, the sensitivity of RDTs typically ranges from 0.78 to 0.87; thus, we set the RDT sensitivity to 0.8 [23]. In addition, δ represents the allowable error and is set to 0.06; p represents the HBsAg prevalence in Guangdong Province. For the period from 2006 to 2021, the prevalence of HBsAg in Guangdong was 5.7% (95% CI: 4.9–6.7) [5]. To increase the sample size and improve the reliability of the results, we assumed that the HBsAg prevalence rate in Guangdong Province for 2024 was 6.3%. We calculated that the minimum required sample size was 2710, which is smaller than the sample size included in this study.

The inclusion criteria for the study participants were (1) permanent residents who had lived in the local community for at least six months and (2) individuals willing to participate in this study. The exclusion criteria were (1) participants who experienced an acute illness at the time of the survey; (2) an axillary temperature > 37.2 °C; and (3) a history of thrombocytopenia or other coagulation disorders that could serve as contraindications for intramuscular injection.

### 2.2. Demographic and Epidemiological Information Collection

Demographic information was collected through questionnaire surveys. Moreover, venous blood samples were collected from each participant and transported to the Guangdong Provincial Institute of Public Health at 4 °C within 24 h. Venous blood was centrifuged at 3000 rpm for 5 min, and serum was transferred to cryogenic tubes and stored at −80 °C. Maps of Guangdong Province were downloaded from the China National Geographic Information Public Service Platform (https://cloudcenter.tianditu.gov.cn/administrativeDivision, Map Content Approval Number: GS (2024) 0650, accessed on 13 January 2026).

### 2.3. RDT and Laboratory Serological Immunoassay for HBsAg Detection

The HBsAg concentration of each serum sample was qualitatively determined through an RDT (Wondfo Biotech, Guangzhou, China), which utilizes the immune colloidal gold technique. If only the control region shows a red band, the HBsAg-RDT is considered negative. When both the control region and test region show red bands, the HBsAg-RDT is considered positive. For the RDT, the lower limit of detection for HBsAg is 5 IU/mL. Additionally, a laboratory serological immunoassay, which is based on an ECLIA, was used to detect HBsAg quantitatively, and the reagent used was Elecsys HBsAg II quant II reagent (Roche Diagnostics GmbH, Mannheim, Germany), with a minimum detection limit of 0.05 IU/mL. For the ECLIA, the quantitative HBsAg detection range was determined to be 0.05~52,000 IU/mL. Quantitative HBsAg levels < 0.05 IU/mL were considered HBsAg negative, while quantitative HBsAg levels ≥ 0.05 IU/mL were considered HBsAg-ECLIA positive. The detection procedures were implemented according to the instructions. We retested samples that were HBsAg-ECLIA positive. If both results were positive, we considered the HBsAg-ECLIA result to be positive and averaged the HBsAg-ECLIA levels. Otherwise, we considered the HBsAg-ECLIA results to be negative.

### 2.4. NAT for HBV DNA Detection

Total DNA from each serum sample was extracted using a Magnetic Universal Virus DNA/RNA Extraction Kit (Baypure, Guangzhou, China) with a fully automated nucleic acid extractor (Tianlong, Xi’an, China). An HBV nucleic acid detection kit (Daan Gene, Guangzhou, China) was used to assess DNA samples extracted from serum, and a CFX96 Touch Real-Time Quantitative PCR System (Bio-Rad Laboratories, Hercules, CA, USA) was used to measure the level of HBV DNA in the samples. In accordance with the manufacturer’s instructions for the HBV nucleic acid detection kit, a cycle threshold (CT) < 45 was considered positive for HBV DNA, indicating that the lower limit of detection for qualitative detection of HBV DNA is 0. Furthermore, the lower limit of detection for quantitative detection of HBV DNA was 2 × 10^1^~10^9^ IU/mL. Additionally, we retested samples that were HBV DNA-NAT positive. If both results were positive, we considered the HBV DNA-NAT result to be positive and averaged the load of the HBV DNA-NATs. Otherwise, we considered the HBV DNA-NAT result to be negative.

### 2.5. Cost–Benefit Analysis

In this study, the screening costs, number of HBV-infected individuals detected, screening benefits, and cost–benefit ratio (CBR) per 100,000 people for implementing the RDT, NAT, or RDT and NAT in parallel in Guangdong were calculated. Additionally, we calculated the additional cost–benefit ratio (ACBR) for switching from the RDT to the NAT or the parallel RDT and NAT in Guangdong. If the ACBR is <1, switching from the RDT to the NAT or RDT and NAT in parallel is cost effective; otherwise, it is not cost effective. The calculation process for each indicator is as follows:

Screening cost: Screening cost = number of samples × cost per sample test × reagent efficiency. For each sample, the cost of conducting an RDT is $1.15, that of an NAT is $3.35, and that of an RDT in parallel with an NAT is $4.50. The cost per sample test includes reagent expenses alone. The reagent efficiency was set at 1.05 [24].

Number of HBV-infected individuals detected: Number of HBV-infected individuals detected = number of samples × HBsAg-ECLIA incidence × sensitivity of screening method. The number of samples was set to 100,000.

Screening benefit: Screening Benefit = Number of HBV-infected individuals detected × Average net benefit per HBV-infected individual detected. According to Su’s study, on the basis of the 2021 baseline and predictions for a five-test screening program among individuals aged 18~70, the average net monetary benefit (NMB) per HBV-infected individual detected is $442 (Table S1) [21].

CBR: CBR = Screening cost/screening benefit.

ACBR: ACBR = (Screening cost of NAT/RDT + NAT—Screening cost of RDT)/(Screening benefit of NAT/RDT + NAT—Screening benefit of RDT) [24].

### 2.6. Statistical Analysis

Medians and interquartile ranges were used to describe the central tendency and dispersion tendency of the HBsAg and HBV DNA levels. The Wilson–Brown method was used to calculate 95% confidence intervals (CIs) for the rates. The chi-square test or Fisher’s exact test was used to analyze differences in prevalence. The Wilcoxon rank-sum test was used to compare the distributions of quantitative data between two groups. The McNemar test was used to analyze differences in the qualitative results of two hepatitis B screening methods for the same individual. Polynomial logistic regression was used to analyze the impact of regional GDP on HBsAg-ECLIA prevalence, adjusting for sex, age (linear term), and age trend (quadratic term). According to the Guangdong Statistical Yearbook 2025, in 2024, the sex distribution of the population was 52.6% male and 47.4% female. The age distribution was 17.5% aged 0~14, 72.3% aged 15~64, and 10.2% aged ≥ 65 years. The distributions of permanent residents of Guangzhou, Qingyuan, and Heyuan were 73.5%, 15.5%, and 11.0%, respectively. Direct standardization was used to adjust the HBsAg-ECLIA prevalence.

In this study, the screening performances of the RDT and NAT and the RDT and NAT in parallel were evaluated using an ECLIA as the gold standard. For the RDT and NAT in parallel, if either the RDT or NAT was positive, the parallel RDT and NAT was considered positive; otherwise, it was considered negative. The screening performance parameters included sensitivity, specificity, positive predictive value (PPV), negative predictive value (NPV), and kappa value. For the RDT, HBsAg-ECLIA positivity/HBsAg-RDT positivity was considered a true positive, HBsAg-ECLIA positivity/HBsAg-RDT negativity was considered a false negative, HBsAg-ECLIA negativity/HBsAg-RDT positivity was considered a false positive, and HBsAg-ECLIA negativity/HBsAg-RDT negativity was considered a true negative. Sensitivity was defined as the proportion of true positives among HBsAg-ECLIA-positive results, and specificity was defined as the proportion of true negatives among HBsAg-ECLIA-negative results. The prevalence rate of the Bayesian-corrected HBsAg-ECLIA results was used to calculate the PPV and NPV. The kappa value was calculated to demonstrate the correlation with the HBsAg-ECLIA results. A kappa value > 0.75 indicated a good correlation with the HBsAg-ECLIA, and a kappa value < 0.4 indicated a poor correlation with the HBsAg-ECLIA. Additionally, for the NAT, sensitivity was defined as the proportion of HBsAg-ECLIA-positive/HBV DNA-NAT-positive results among HBsAg-ECLIA-positive results, and specificity was defined as the proportion of HBsAg-ECLIA-negative/HBV DNA-NAT-negative results among HBsAg-ECLIA-negative results. For the parallel RDT and NAT, sensitivity was defined as the proportion of HBsAg-ECLIA-positive/RDT + NAT-positive results among the HBsAg-ECLIA-positive results, and specificity was defined as the proportion of HBsAg-ECLIA-negative/RDT + NAT-negative results among the HBsAg-ECLIA-negative results. We calculated the sensitivity, specificity, PPV, NPV and kappa value of the NAT and the RDT and NAT in parallel using the same approach. Statistical significance was set at 0.05. Statistical analysis was performed using R 4.4.2.

## 3. Results

### 3.1. Demographic Information

A total of 2721 participants were recruited for this study. In terms of sex, 1294 participants were male (47.6%) and 1427 were female (52.4%). The median age of the participants was 20.0 years (IQR: 4.6–47.0). The ages of the participants ranged from 0 to 97 years. The largest age group was 1~4 years (381 participants, 14.0%), while the smallest was 70 years and older (167 participants, 6.1%). In terms of region, 742 participants (27.3%) were from Guangzhou, 981 (36.1%) were from Qingyuan, and 998 (36.6%) were from Heyuan (Table 1).

### 3.2. Prevalence Rates of the HBsAg-ECLIA, HBsAg-RDT and HBV DNA-NAT

**Figure 1:**
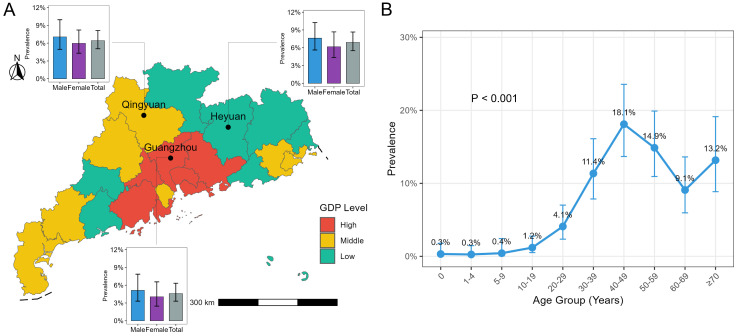
Gross domestic product (GDP) and HBsAg-ECLIA prevalence rates in Guangdong Province in 2024. (**A**) GDP distribution across Guangdong Province and HBsAg-ECLIA prevalence stratified by region and sex. Red, yellow, and green represent high, medium, and low GDP levels, respectively. Bar charts depict HBsAg-ECLIA prevalence stratified by region and sex. (**B**) HBsAg-ECLIA prevalence stratified by age group. The chi-square test was used to analyze differences in HBsAg-ECLIA prevalence across age groups.

Among the 2721 participants, 166 HBsAg-ECLIA-positive samples were included in this study, with a prevalence of 6.10% (95% CI: 5.26~7.06). After adjustment for sex, age, and regional population weighting, the standardized prevalence of HBsAg was 5.48% (95% CI: 4.97~5.99) in selected representative regions of Guangdong. The prevalence rate of the HBsAg-ECLIA was 6.72% (95% CI: 5.48~8.22) in males and 5.54% (95% CI: 4.46~6.85) in females. With respect to region, the prevalence rates of the HBsAg-ECLIA in Guangzhou, Qingyuan, and Heyuan were 4.58% (95% CI: 3.30~6.33), 6.42% (95% CI: 5.05~8.13), and 6.91% (95% CI: 5.50~8.66), respectively (Figure 1A). For all age groups, the lowest prevalence of the HBsAg-ECLIA was observed among those aged 1~4 years (0.26%; 95% CI: 0.01~1.47), while the highest prevalence rate was among those aged 40~49 years (18.10%; 95% CI: 13.68~23.56). Since the hepatitis B vaccine was included in the national immunization program in 1992, the incidence of HBV infection has decreased significantly among newborns born after 1992 (approximately 0~29 years of age). Therefore, we focused on the prevalence of HBsAg and HBV DNA in the 0~29-year-old group and the 30-year-old and above group. The prevalence rate of the HBsAg-ECLIA among individuals aged 0~29 years was 1.23% (95% CI: 0.80–1.89), and among those aged 30 years and older, it was 11.18% (95% CI: 9.53~13.08). In terms of the HBsAg-RDT and HBV DNA-NAT results, 135 HBsAg-RDT-positive samples and 177 HBV DNA-NAT-positive samples were included in this study, resulting in prevalence rates of 4.96% (95% CI: 4.21~5.84) and 6.50% (95% CI: 5.64~7.49), respectively. The prevalence rates of the HBsAg-RDT and HBV DNA-NAT among individuals aged 0~29 years were 0.98% (95% CI: 0.60~1.59) and 2.45% (95% CI: 1.81~3.32), respectively, whereas among those aged 30 years and older, they were 10.92% (95% CI: 9.20~12.91) and 12.57% (95% CI: 10.73~14.67, Table S2), respectively. Overall, the prevalence rate of the HBsAg-ECLIA exhibited an inverted U-shaped trend (Figure 1B). After adjusting for the effects of sex, age, and a nonlinear trend of age using polynomial logistic regression, the regional GDP was revealed to be an independent predictor of HBsAg prevalence, which indicates that regions with higher economic levels have lower HBsAg prevalence (OR = 0.796, 95% CI: 0.640~0.987; Table S3).

### 3.3. Characteristics of HBsAg-ECLIA-Negative/HBV DNA-NAT-Positive and HBsAg-ECLIA-Positive/HBV DNA-NAT-Negative Samples

**Figure 2:**
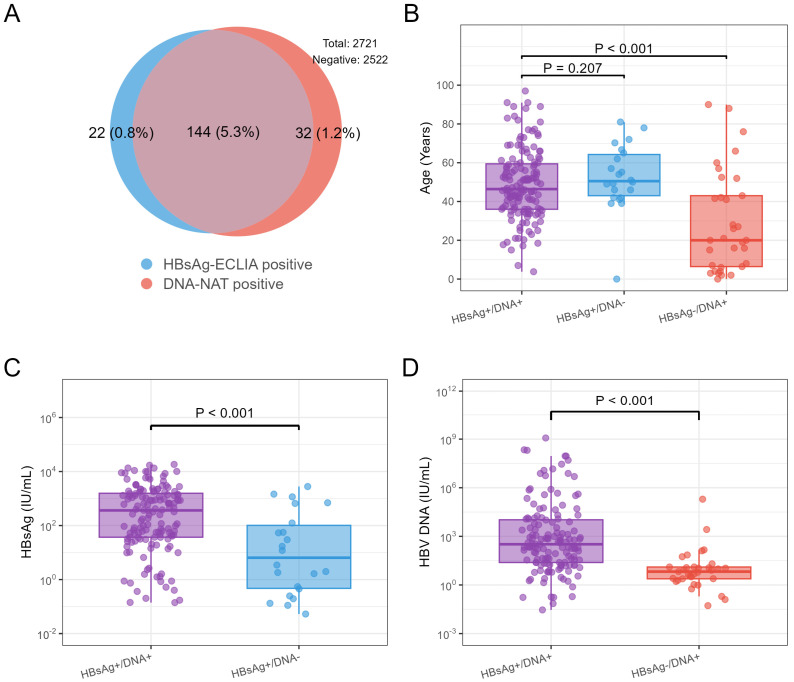
Characteristics of HBsAg-ECLIA-negative/HBV DNA-NAT-positive and HBsAg-ECLIA-positive/HBV DNA-NAT-negative participants. (**A**) Euler diagram illustrating the overlap between HBsAg-ECLIA-positive and DNA-NAT-positive participants. The numbers in circles indicate participant counts; the percentages in parentheses indicate the proportions of all participants. The upper right corners represent the total number of participants and the number of HBsAg-ECLIA-negative/HBV DNA-NAT-negative participants. (**B**) Age differences among HBsAg-ECLIA-positive/HBV DNA-NAT-positive, HBsAg-ECLIA-positive/HBV DNA-NAT-negative, and HBsAg-ECLIA-negative/HBV DNA-NAT-positive participants. (**C**) Comparison of HBsAg-ECLIA levels between HBsAg-ECLIA-positive/HBV DNA-NAT-positive and HBsAg-ECLIA-positive/HBV DNA-NAT-negative participants. (**D**) Comparison of HBV DNA-NAT loads between HBsAg-ECLIA-positive/HBV DNA-NAT-positive and HBsAg-ECLIA-negative/HBV DNA-NAT-positive participants. Wilcoxon rank-sum tests were used to analyze differences in age, HBsAg-ECLIA levels, and HBV DNA-NAT loads.

Among the healthy individuals from the community, 144 were HBsAg-ECLIA positive/HBV DNA-NAT positive, accounting for 5.3% of the total participants (95% CI: 4.5~6.2). There were 22 HBsAg-ECLIA-positive/HBV DNA-NAT-negative participants, accounting for 0.8% of the total participants (95% CI: 0.5~1.2). There were 32 HBsAg-ECLIA-negative/HBV DNA-NAT-positive participants, accounting for 1.2% of the total participants (95% CI: 0.9~1.7; Figure 2A). Moreover, the percentage of HBsAg-ECLIA-negative/HBV DNA-NAT-positive individuals whose HBV DNA concentration was less than 200 IU/mL was 1.1% in this study (95% CI: 0.8~1.6). Among individuals who were HBsAg-ECLIA positive/HBV DNA-NAT positive, HBsAg-ECLIA positive/HBV DNA-NAT negative, and HBsAg-ECLIA negative/HBV DNA-NAT positive, 86.8% (125/144), 95.5% (21/22), and 37.5% (12/32), respectively, were aged 30 years and above. Moreover, the age of the HBsAg-ECLIA-negative/HBV DNA-NAT-positive participants was significantly younger than that of the HBsAg-ECLIA-positive/HBV DNA-NAT-positive participants (*p* < 0.001; Figure 2B). We further compared HBsAg levels between HBsAg-ECLIA-positive/HBV DNA-NAT-positive and HBsAg-ECLIA-positive/HBV DNA-negative participants and reported significantly higher HBsAg ECLIA levels in HBsAg-ECLIA-positive/HBV DNA-NAT-positive participants (*p* < 0.001; Figure 2C). Similarly, the HBV DNA loads of HBsAg-ECLIA-positive/HBV DNA-NAT-positive participants were significantly greater than those of HBsAg-ECLIA-negative/HBV DNA-NAT-positive participants, and 81.3% (26/32) of the HBsAg-ECLIA-negative/HBV DNA-NAT-positive participants exhibited HBV DNA loads less than 20 IU/mL (*p* < 0.001; Figure 2D).

### 3.4. Evaluation of the Screening Effectiveness for Hepatitis B of the HBsAg-RDT and HBV DNA-NAT

Using the ECLIA as the gold standard, this study demonstrated the screening performance of the RDT, NAT, and RDT and NAT in parallel. In terms of the RDT, the sensitivity was 80.7% (95% CI: 74.1~86.0), the specificity was 100.0% (95% CI: 99.8~100.0), and the kappa value was 0.884 (95% CI: 0.845~0.923). We compared HBsAg-ECLIA levels between true-positive and false-negative RDT results. The median HBsAg concentration among true-positive participants was 631.0 IU/mL (IQR: 61.0~1805.5). Among the false-negative participants, 87.5% (28/32) had HBsAg-ECLIA levels less than 5 IU/mL, and the median HBsAg concentration was 0.862 IU/mL (IQR: 0.236–1.860), which was significantly lower than that of the true-positive participants (*p* < 0.001; Figure S1). For the NAT, the sensitivity was 86.7% (95% CI: 80.7~91.1), the specificity was 98.7% (95% CI: 98.2~99.1), and the kappa value was 0.829 (95% CI: 0.785~0.873). For the RDT and NAT in parallel, the sensitivity was 93.4% (95% CI: 88.5~96.6), the specificity was 98.7% (95% CI: 98.2~99.1), and the kappa value was 0.867 (95% CI: 0.828~0.906). No significant difference in sensitivity was observed between the NAT and RDT (*p* = 0.112). Nevertheless, the sensitivity of the parallel RDT and NAT was significantly greater than that for the RDT alone (*p* < 0.001; Table 2).

### 3.5. Cost–Benefit Analysis of Screening Strategies for Hepatitis B

For screening 100,000 individuals as an example, the screening costs for the RDT, NAT, and RDT and NAT in parallel were $120,645.5, $352,888.0, and $473,533.4, respectively. The numbers of HBV-infected individuals detected were 4422.4, 4751.2, and 5118.3, respectively. The screening benefits were $1,954,700.8, $2,100,030.4, and $2,262,288.6, respectively. The CBRs were 1:16.2, 1:6.0, and 1:4.8, respectively. Nevertheless, compared with the RDT, both the NAT and the RDT and NAT in parallel resulted in an ACBR > 1, with values of 1:0.6 and 1:0.9, respectively, which indicates that switching from the RDT to the NAT or the RDT and NAT in parallel in the general population is not cost effective (Table S4). After stratification by sex, age, and region, we further analyzed the number of HBV-infected individuals detected, the CBR, and the ACBR. We found that the number of HBV-infected individuals detected and the CBR varied with age. The lowest number of HBV-infected individuals detected and the highest CBR were observed in the 1~4-year age group. The numbers of HBV-infected individuals detected for the RDT, NAT, and RDT and NAT in parallel were 209.82, 225.42, and 242.84, respectively, while the CBRs were 1:0.77, 1:0.28, and 1:0.23, respectively. The highest number of HBV-infected individuals detected and the lowest CBR were observed in the 40~49 age group. The numbers of HBV-infected individuals detected by the RDT, NAT, and RDT and NAT in parallel were 14,606.70, 15,692.70, and 16,905.40, respectively, while the CBRs were 1:53.51, 1:19.66, and 1:15.78, respectively (Table 3). Compared with the RDT, the NAT had an ACBR < 1 in individuals aged 30 years and older, and the RDT and NAT in parallel had an ACBR < 1 in males, individuals aged 30 years and older, and those in Qingyuan and Heyuan. For each stratum, compared with that of the RDT, the ACBR of adjusting the screening strategy to the RDT and NAT in parallel was lower than that of adjusting to the strategy of the NAT alone, which indicates that switching the RDT screening strategy to the RDT and NAT in parallel is more cost effective than switching to the NAT alone. (Table 4).

## 4. Discussion

In accordance with the findings of this study, the standardized prevalence rate of the HBsAg-ECLIA in the general population of selected areas in Guangdong Province was 5.48% in 2024. This represents a decrease compared with the overall HBsAg prevalence of 5.7% observed in Guangdong Province between 2006 and 2021 [5]. The prevalence rate of the HBsAg-ECLIA among individuals aged 30 years and younger was less than 1.23%. These findings confirm the remarkable success of Guangdong in preventing mother-to-child transmission and establishing population immunity barriers after the hepatitis B vaccine was incorporated into the immunization program in 1992 [25]. Nevertheless, there is a persistently substantial hepatitis B disease burden in Guangdong [6]. In this study, the HBsAg-ECLIA prevalence exhibited an inverted U-shaped distribution with increasing age, which is consistent with the findings of previous studies [26]. The prevalence rate of the HBsAg-ECLIA peaked at 18.10% among individuals aged 40~49 years. This is because individuals in the 40~49-year-old age group were born before widespread vaccination against hepatitis B and therefore constitute the majority of the hepatitis B disease burden [3,27]. Furthermore, after adjusting for the effects of demographic characteristics using polynomial logistic regression, we found that regional GDP was an independent predictor of the HBsAg-ECLIA prevalence rate. The prevalence rate of the HBsAg-ECLIA was higher in regions with lower GDP levels than in those with higher GDP levels. These findings indicate that the HBsAg-ECLIA prevalence is concentrated in regions with lower GDPs [28,29,30].

In this study, the participants were healthy individuals from the community, including all the HBV-infected individuals. Demirturk et al. reported that inactive HBV carriers, who are defined as HBsAg-positive individuals who are negative for HBV DNA or have low-level HBV DNA, constitute the largest population of HBV-infected individuals [31]. Chen et al. [32] conducted a community-based epidemiological survey in Zhejiang Province, China, defining participants with HBV DNA levels <1000 copies/mL (approximately 178 IU/mL) as negative. Among 8439 HBsAg-positive individuals, approximately 46% were negative for HBV DNA. For our study, we defined negativity as a cycle threshold (CT) ≥45, and the proportion of HBsAg-positive/HBV DNA-negative individuals in our study was 0.8%. If an HBV DNA concentration < 178 IU/mL is considered negative, the proportion of HBsAg-positive/HBV DNA-negative individuals in this study was 49.4%, which is similar to the results reported by Chen. Additionally, OBI is typically defined as HBsAg negativity and HBV DNA positivity. Since acute HBV infection is also typically characterized by HBsAg-negative and HBV DNA-positive results during the window period, the above definition of OBI could not distinguish between OBI and acute HBV infection. Given that the HBV DNA load in OBI is typically less than 200 IU/mL, whereas the HBV DNA load in acute HBV infection during the window period is greater than 200 IU/mL, an increasing number of researchers recommend defining OBI as HBsAg negativity, HBV DNA positivity, and an HBV DNA load of less than 200 IU/mL. Therefore, to exclude patients with acute HBV infection during the window period, this study defined OBI as HBsAg negativity, HBV DNA positivity, and an HBV DNA load of less than 200 IU/mL [33,34]. The prevalence of OBI among healthy individuals in this community-based study was 1.1%. Ji et al. analyzed the OBI prevalence in healthy populations and reported that the prevalence was 0.88% (95% CI: 0.55~1.27) in China [35]. These findings indicate that the OBI prevalence in this study is consistent with findings from other researchers. Nevertheless, Ye’s study participants were drawn from the donor population of a single blood center in Guangdong, not the general community across all age groups. Furthermore, the prevalence of HBsAg in Ye’s study was 0.61%, which was lower than the average prevalence of HBsAg in Guangdong Province from 2006 to 2021 (5.7%) [5,36]. This is attributed to the fact that blood donation centers often use an RDT to prescreen donors, excluding those who test positive for HBsAg via the RDT, and then conduct further HBsAg and HBV DNA testing on the remaining pool of donors, which significantly reduces the prevalence of HBsAg and HBV DNA among blood donors. In addition, blood donors tend to be younger and often have a history of hepatitis B vaccination, which also contributes to the lower probability of HBV infection. Su et al. conducted a cohort study of 0.6 million blood donors in China and reported a healthy donor effect among blood donors, which indicates that the disease incidence among blood donors is significantly lower than that among nonblood donors [37]. These findings indicate that the healthy donor effect among blood donors significantly underestimates the prevalence of HBsAg. Therefore, using blood donors as a representative sample of the community population would significantly underestimate the prevalence of HBsAg and other HBV infection statuses, including inactive HBV carriers and OBI. According to the findings shown in Figure 2B, compared with the HBsAg-positive/HBV DNA-positive participants, individuals with OBI were younger. This is likely due to persistent low-level viral carriage following mother-to-child transmission, particularly among younger generations who were vaccinated extensively against hepatitis B [38]. Moreover, among the HBsAg-positive/HBV DNA-negative individuals from the community-based healthy population in this study, 95.5% were aged 30 years or older. Ren et al. conducted a multicenter retrospective study and reported that the proportion of HBsAg-positive/DNA-negative individuals (those who had not received antiviral treatment) aged 30 years or older was 94.0%, which is consistent with the results of our study [39]. Moreover, our study revealed significantly higher HBsAg levels in HBsAg-positive/HBV DNA-positive participants than in HBsAg-positive/HBV DNA-negative participants (*p* < 0.001). HBsAg-positive/HBV DNA-negative status indicates low or inactive HBV activity, which is correlated with a decreased risk of disease progression. Therefore, the WHO and the U.S. Centers for Disease Control and Prevention recommend regular monitoring of alanine aminotransferase levels and HBV DNA loads in HBsAg-positive/HBV DNA-negative individuals, without the need for antiviral therapy [17,40]. However, a study by Tang [41] indicated that OBI is associated with cirrhosis, which suggests the need to enhance the detection and follow-up of individuals with OBI, thereby reducing the harm caused by cirrhosis. Additionally, the HBV DNA loads in individuals with OBI were lower than those in HBsAg-positive/HBV DNA-positive individuals (*p* < 0.001), and 81.3% of the individuals with OBI had HBV DNA loads less than 20 IU/mL, which was consistent with the findings of other studies [42,43].

It is challenging to meet the requirements for accurate diagnosis and elimination of hepatitis B using an RDT alone [44]. According to the findings of this study, with an ECLIA as the gold standard, the sensitivity of the RDT was 80.7%. This suggests that implementing an RDT for screening in the real world would result in approximately 20% of HBsAg-ECLIA-positive individuals being undetected. Jargalsaikhan’s study reported 100.0% sensitivity for Wondfo’s HBsAg-RDT, which is the same reagent evaluated in this study. Nevertheless, Jargalsaikhan’s study used an enzyme-linked immunosorbent assay as the gold standard, potentially overestimating RDT sensitivity by neglecting low-level HBsAg-ECLIA results [45,46]. According to the results of our study, there was no significant difference in sensitivity between the NAT and RDT (*p* = 0.112). Further analysis revealed that HBsAg-ECLIA levels in the RDT false-negative participants were significantly lower than those in the true-positive participants, which confirmed the limitation of RDT performance in detecting low-concentration HBsAg samples [47]. Nevertheless, adopting an NAT alone as a screening strategy for hepatitis B did not significantly increase the number of HBsAg-positive individuals detected, and the kappa value was lower than that of the RDT alone. Multiple factors contribute to this outcome, including differences in the biological markers detected by the ELCIA and NAT, as well as the presence of diverse HBV infection statuses among the community-based healthy population. The effectiveness of screening methods in this study was evaluated using a community-based healthy population that had various HBV infection statuses rather than solely focusing on HBsAg+/HBV DNA+ individuals. Among community-based healthy individuals, a significant proportion are inactive HBV carriers (HBsAg+/HBV DNA−) and have OBI (HBsAg-/HBV DNA+). OBI cannot be detected by HBsAg testing alone, and inactive HBV carriers cannot be detected by HBV DNA testing alone. Consequently, this results in lower NAT sensitivity and kappa values when an ECLIA is used as the gold standard for the NAT in healthy community-based populations [31,35,48]. Therefore, we evaluated the RDT and NAT in parallel as a hepatitis B screening strategy. The sensitivity of the RDT and NAT in parallel was significantly greater than that for the RDT alone (*p* < 0.001). This finding indicates that compared with the RDT alone, the RDT and NAT in parallel can increase true-positive results [46,49].

In our study, the CBRs for the NAT and the RDT and NAT in parallel were less than 1, but the ACBR exceeded 1 in the general population, which indicates that switching from the RDT to the NAT or RDT and NAT in parallel in the general population is not cost effective [50]. After stratification by age, the RDT and NAT in parallel revealed an ACBR < 1 for individuals aged 30 years and older. Compared with the RDT alone, implementing the RDT and NAT in parallel in individuals aged 30 years and older will result in greater economic benefits [51]. When stratified by region, the ACBR for the RDT and NAT in parallel was less than 1 in Qingyuan and Heyuan. Consequently, compared with the RDT alone, implementing the RDT and NAT in parallel in Qingyuan and Heyuan will result in greater economic benefits. Nevertheless, Qingyuan and Heyuan represent medium- and low-GDP regions, respectively, and evaluating whether the regional economy is sufficient to implement the RDT and NAT in parallel is necessary [21].

There are certain limitations to our study. Compared with those in other community-based studies, the sample size in this study is relatively small; thus, increasing the sample size is needed in future research [32,52]. Additionally, in this study, the average NMB per HBV-infected individual identified in the 18~70 age group was used in the general population. The average NMB per HBV-infected individual detected may vary between the 18~70 age group and those aged 18 and younger [53]. Finally, we detected only HBsAg and HBV DNA, not anti-HBc antibodies, which assisted in distinguishing between window period infection and true OBI in HBsAg-positive/HBV DNA-positive individuals [54,55].

## 5. Conclusions

In 2024, the standardized prevalence rate of the HBsAg-ECLIA in certain regions of Guangdong was 5.48%, and adults aged 30 years and older composed the main hepatitis B infection population, with an HBsAg-ECLIA prevalence rate of 11.18%. Using the RDT as the primary hepatitis B screening strategy will result in individuals with low-level HBsAg being missed. To achieve the WHO’s 2030 hepatitis B elimination target and improve the economic benefits of hepatitis B screening, it is recommended that Guangdong Province implement an RDT for individuals aged 30 and younger and the RDT and NAT in parallel for individuals aged 30 and older.

## Figures and Tables

**Table 1:** Demographic characteristics and number of participants.

Characteristics		Number (%)
Sex		
	Male	1294 (47.6)
	Female	1427 (52.4)
Age group		
	0	311 (11.4)
	1~4	381 (14.0)
	5~9	229 (8.4)
	10~19	417 (15.3)
	20~29	293 (10.8)
	30~39	229 (8.4)
	40~49	232 (8.5)
	50~59	242 (8.9)
	60~69	220 (8.1)
	70 years old and above	167 (6.1)
Region		
	Guangzhou	742 (27.3)
	Qingyuan	981 (36.1)
	Heyuan	998 (36.6)
Total		2721 (100.0)

**Table 2:** Performance of hepatitis B screening strategies.

Strategies	Sensitivity ^a^ (%, 95% CI)	Specificity ^b^ (%, 95% CI)	PPV ^c^ (%, 95% CI)	NPV ^d^ (%, 95% CI)	Kappa Value ^e^ (95% CI)	*p* Value ^f^
RDT	80.7 (73.9~86.4)	100 (99.8~100)	99.3 (95.9~100)	98.8 (98.3~99.2)	0.884 (0.845~0.923)	/
NAT	86.7 (80.6~91.5)	98.7 (98.2~99.1)	81.4 (74.8~86.8)	99.1 (98.7~99.5)	0.829 (0.785~0.873)	0.112
RDT + NAT ^g^	93.4 (88.5~96.6)	98.7 (98.2~99.1)	82.4 (76.2~87.6)	99.6 (99.2~99.8)	0.867 (0.828~0.906)	<0.001

^a^—Sensitivity was defined as the proportion of HBsAg-ECLIA-positive/HBsAg-RDT-positive results for the RDT, HBsAg-ECLIA-positive/HBV DNA-NAT-positive results for the NAT, and HBsAg-ECLIA-positive/RDT + NAT-positive results for the RDT and NAT in parallel among the HBsAg-ECLIA-positive results. ^b^—Specificity was defined as the proportion of HBsAg-ECLIA-negative/HBsAg-RDT-negative results for the RDT, HBsAg-ECLIA-negative/HBV DNA-NAT-negative results for the NAT, and HBsAg-ECLIA-negative/RDT + NAT-negative results for the RDT and NAT in parallel among the HBsAg-ECLIA-negative results. ^c^—PPV, positive predictive value. ^d^—NPV, negative predictive value. ^e^—A kappa value > 0.75 indicates good consistency with the ECLIA; a kappa value < 0.4 indicates poor consistency with the ECLIA. ^f^—The McNemar test was used to analyze the differences in sensitivity between the RDT and NAT, as well as between the RDT and the RDT and NAT in parallel. ^g^—RDT + NAT, RDT and NAT in parallel.

**Table 3:** Number of HBV-infected individuals detected and the cost–benefit ratio (CBR) for the three screening strategies.

Characteristics		RDT		NAT		RDT + NAT ^b^	
		*n* ^a^	CBR	*n*	CBR	*n*	CBR
Sex							
	Male	5423.04	1:19.87	5826.24	1:7.30	6276.48	1:5.86
	Female	4470.78	1:16.38	4803.18	1:6.02	5174.36	1:4.83
Age group							
	0	258.24	1:0.95	277.44	1:0.35	298.88	1:0.28
	1~4	209.82	1:0.77	225.42	1:0.28	242.84	1:0.23
	5~9	355.08	1:1.30	381.48	1:0.48	410.96	1:0.38
	10~19	968.40	1:3.55	1040.40	1:1.30	1120.80	1:1.05
	20~29	3308.70	1:12.12	3554.70	1:4.45	3829.40	1:3.57
	30~39	9159.45	1:33.56	9840.45	1:12.33	10,600.90	1:9.89
	40~49	14,606.70	1:53.51	15,692.70	1:19.66	16,905.40	1:15.78
	50~59	12,008.16	1:43.99	12,900.96	1:16.16	13,897.92	1:12.97
	60~69	7335.63	1:26.88	7881.03	1:9.87	8490.06	1:7.92
	70 years old and above	10,628.19	1:38.94	11,418.39	1:14.30	12,300.78	1:11.48
Region							
	Guangzhou	3696.06	1:13.54	3970.86	1:4.97	4277.72	1:3.99
	Qingyuan	5180.94	1:18.98	5566.14	1:6.97	5996.28	1:5.60
	Heyuan	5576.37	1:20.43	5990.97	1:7.50	6453.94	1:6.02

^a^—*n* represents the number of HBV-infected individuals detected in a population of 100,000 individuals. ^b^—RDT + NAT, RDT and NAT administered in parallel.

**Table 4:** Additional cost–benefit ratio (ACBR) ^a^ of the NAT and the RDT and NAT in parallel compared with that of the RDT.

Characteristics		NAT	RDT + NAT ^b^
Sex			
	Male	1:0.77	1:1.07
	Female	1:0.63	1:0.88
Age group			
	0	1:0.04	1:0.05
	1~4	1:0.03	1:0.04
	5~9	1:0.05	1:0.07
	10~19	1:0.14	1:0.19
	20~29	1:0.47	1:0.65
	30~39	1:1.30	1:1.81
	40~49	1:2.07	1:2.88
	50~59	1:1.70	1:2.37
	60~69	1:1.04	1:1.45
	70 years old and above	1:1.50	1:2.09
Region			
	Guangzhou	1:0.52	1:0.73
	Qingyuan	1:0.73	1:1.02
	Heyuan	1:0.79	1:1.10

^a^—Data are presented as the ACBR (1: X). A value of X > 1 indicates that the economic benefit exceeds the investment cost, which is equal to an ACBR < 1. A value of X < 1 indicates that the economic benefit does not exceed the investment cost, which is equal to an ACBR > 1. ^b^—RDT + NAT, RDT and NAT administered in parallel.

## Data Availability

Data is provided within the manuscript. Further inquiries can be directed to the corresponding authors.

## Supplementary Materials

The following supporting information can be downloaded at https://www.mdpi.com/article/10.3390/v18050486/s1, Table S1: Calculation process for the average net monetary benefit (NMB) per HBV-infected individual detected, Table S2: Prevalence of HBsAg-ECLIA, HBsAg-RDT and HBV DNA-NAT, Table S3: Multivariate Analysis of HBsAg-ECLIA Prevalence, Table S4: Cost–benefit analysis of three screening strategies, Figure S1: Comparison of HBsAg-ECLIA levels between true-positive and false-negative participants of HBsAg-RDT.
