# Supplementary material for: Characteristics and Screening Strategies of Hepatitis B in Guangdong Province, China
Source: Viruses. 2026 Apr 22;18(5):486. doi: 10.3390/v18050486 (PMC13211652; doi:10.3390/v18050486)
Supplement: Supplementary file 1 [file viruses-18-00486-s001.zip › viruses-4218382-supplementary.pdf]

Table S1. Calculation process for the average net monetary benefit (NMB) per HBV-infected individual detected[a]

Parameter	Formula		Result
1. Calculate the incremental number of HBV-infected individuals detected			
Number of diagnosed HBV-infected individuals under status quo strategy	= Number of individuals × Individuals living with HBV diagnosed over the lifetime under status quo strategy	= 100000 × 18.9%	1217
Number of diagnosed HBV-infected individuals under five-test strategy	= Number of individuals × Individuals living with HBV diagnosed over the lifetime under five-test strategy	= 100000 × 86.8%	5587
Incremental number of HBV-infected individuals detected	= Number of diagnosed HBV-infected individuals under five-test strategy - Number of diagnosed HBV-infected individuals under status quo strategy	= 5587 - 1217	4370
2. Calculate the NMB of HBV-infected individuals detected			
Incremental investment cost for HBV treatment	= Investment cost for HBV treatment under five-test strategy –Investment cost for HBV treatment under status quo strategy	= 15370000 - 13603000	1767000
Incremental investment cost for screening	= Investment cost for screening under five-test strategy –Investment cost for screening under status quo strategy	= 1066000 - 267000	799000

Total incremental investment cost for HBV-infected individuals	= Incremental investment cost for HBV treatment + Incremental investment cost for screening + Incremental investment cost for vaccination	= 1767000 + 799000 + 253000	2819000
Disability-adjusted life year (QALY) gained through five-test strategy	= Total incremental investment cost for HBV-infected individuals ÷ Incremental cost effectiveness ratio under five-test strategy	= 2819000 ÷ 18295	154
NMB of HBV-infected individuals detected	= (Willingness-to-pay threshold [b] × QALY gained through five-test strategy) - Total incremental investment cost for HBV-infected individuals	= (30828 × 154) - 2819000	1928512
3.Calculate the average NMB per HBV-infected individual detected			
Average NMB per HBV-infected individual detected	= NMB of HBV-infected individuals detected ÷ Incremental number of HBV-infected individuals detected	= 1928512 ÷ 4370	442

a According to Su's study[1], calculations were performed using data from five test strategy and status quo strategy among individuals aged 18 to 70 in 2021. Units for cost and NMB are in US dollars. Units for population is in 100,000 people.

b The willingness-to-pay threshold was set at $30,828 per QALY, which is three times greater than China's per capita gross domestic product in 2019.

Table S2. Prevalence of HBsAg-ECLIA, HBsAg-RDT and HBV DNA-NAT

Characteristics	HBsAg-ECLIA		HBsAg-RDT		HBV DNA-NAT	
	Prevalence	95%CI	Prevalence	95%CI	Prevalence	95%CI

		(%，n/N)[a]		(%，n/N)[a]		(%，n/N)[a]	
Gender							
	Male	6.72 (87/1294)	5.48~8.22	5.41 (70/1294)	4.30~6.78	7.11 (92/1294)	5.83~8.64
	Female	5.54 (79/1427)	4.46~6.85	4.56 (65/1427)	3.59~5.76	5.96 (85/1427)	4.84~7.31
Age group							
	0	0.32 (1/311)	0.02~1.80	0 (0/311)	0~1.22	0.32 (1/311)	0.02~1.80
	1~4	0.26 (1/381)	0.01~1.47	0.26 (1/381)	0.01~1.47	1.84 (7/381)	0.89~3.74
	5~9	0.44 (1/229)	0.02~2.43	0.44 (1/229)	0.02~2.43	2.18 (5/229)	0.94~5.01
	10~19	1.20 (5/417)	0.51~2.78	0.96 (4/417)	0.37~2.44	2.16 (9/417)	1.14~4.05
	20~29	4.10 (12/293)	2.36~7.02	3.41 (10/293)	1.86~6.17	6.14 (18/293)	3.92~9.50
	30~39	11.35 (26/229)	7.87~16.12	10.48 (24/229)	7.14~15.12	10.48 (24/229)	7.14~15.12
	40~49	18.10 (42/232)	13.68~23.56	15.09 (35/232)	11.05~20.26	16.81 (39/232)	12.55~22.15
	50~59	14.88 (36/242)	10.94~19.91	11.57 (28/242)	8.13~16.21	14.05 (34/242)	10.23~18.99
	60~69	9.09 (20/220)	5.96~13.62	8.18 (18/220)	5.24~12.56	8.64 (19/220)	5.60~13.09
	70 years old and above	13.17 (22/167)	8.86~19.14	8.38 (14/167)	5.06~13.58	12.57 (21/167)	8.37~18.46

Region							
	Guangzhou	4.58 (34/742)	3.30~6.33	3.50 (26/742)	2.40~5.08	5.39 (40/742)	3.98~7.26
	Qingyuan	6.42 (63/981)	5.05~8.13	5.40 (53/981)	4.15~7.00	7.24 (71/981)	5.78~9.03
	Heyuan	6.91 (69/998)	5.50~8.66	5.61 (56/998)	4.35~7.22	6.61 (66/998)	5.23~8.33
Total		6.10 (166/2721)	5.26~7.06	4.96 (135/2721)	4.21~5.84	6.50 (177/2721)	5.64~7.49

a n represents the number of participants who tested positive for HBsAg-ECLIA, and N represents the total number of participants.

Table S3. Multivariate Analysis of HBsAg-ECLIA Prevalence[a]

Variable	Odd ratio(95%CI)	P value
Gender	1.55(1.12~2.16)	0.009
Age(linear term))	$6.60\times10^{31}(8.45\times10^{25}\sim3.08\times10^{38})$	＜0.001
Age(quadratic term)	$2.86\times10^{-14}(2.75\times10^{-19}\sim1.08\times10^{-9})$	＜0.001
GDP level	0.796(0.640~0.987)	0.039
Intercept	$3.59\times10^{-2}(2.02\times10^{-2}\sim6.17\times10^{-2})$	＜0.001

a Polynomial logistic regression analysis was used to identify factors influencing the prevalence of HBsAg-ECLIA, with age (quadratic term) incorporated to adjust for the nonlinear trend of age.

Table S4. Cost-benefit analysis of three screening strategies (a population of 100000)

	RDT	NAT	RDT+NAT[a]
Screening cost (Dollar)	120645.5	352888.0	473533.4
Number of HBV-infected individuals detected	4422.4	4751.2	5118.3
Screening benefit (Dollar)	1954700.8	2100030.4	2262288.6

Cost-benefit ratio(CBR)	1：16.2	1：6.0	1：4.8
Additional cost-benefit ratio (ACBR)[b]		1：0.6	1：0.9

a RDT+NAT, RDT and NAT in parallel.

b Data are presented as the ratio of ACBR (1 : X). A value of X > 1 indicates that the economic benefit exceeds the investment cost, which is equal to ACBR < 1. A value of X < 1 indicates that the economic benefit does not exceed the investment cost, which is equal to ACBR > 1.

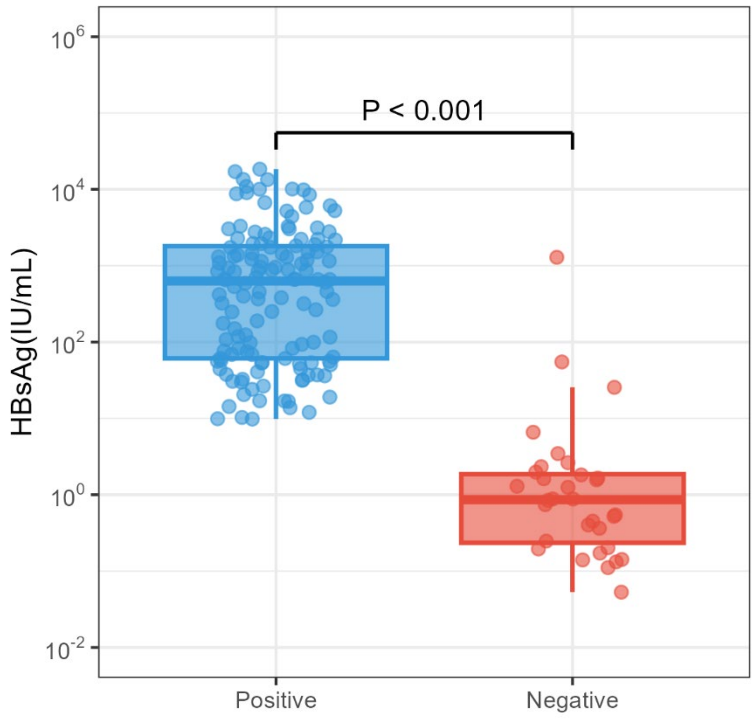

Figure S1. Comparison of HBsAg-ECLIA levels between true positive and false negative participants of HBsAg-RDT. With ECLIA as the gold standard, the left panel shows the HBsAg-RDT true positive participants, and the right panel shows the HBsAg-RDT false negative participants.

Reference

1. Su S, Wong WC, Zou Z, et al. Cost-effectiveness of universal screening for chronic hepatitis B virus infection in China: an economic evaluation. *Lancet Glob Health*. 2022;10(2):e278-e287.
